# Strength and timing of austral winter Angolan coastal upwelling

**DOI:** 10.1038/s41598-024-77917-2

**Published:** 2024-11-09

**Authors:** Mareike Körner, Peter Brandt, Marcus Dengler

**Affiliations:** 1 GEOMAR Helmholtz Centre for Ocean Research Kiel, Kiel, Germany; 2 Present Address: College of Earth, Ocean and Atmospheric Sciences, Oregon State University, Corvallis, OR USA; 3 Faculty of Mathematics and Natural Sciences, Kiel University, Kiel, Germany

**Keywords:** Ocean sciences, Physical oceanography, Physical oceanography

## Abstract

The tropical Angolan upwelling system (tAUS) is a highly productive ecosystem of great socio-economic importance. Productivity peaks in austral winter and is linked to the passage of remotely forced upwelling coastal trapped waves (CTWs), where the strength of the productivity peak is associated with the amplitude of the upwelling CTW. Here, we analyze the year-to-year variability in the timing and amplitude of the austral winter upwelling CTW by examining sea surface temperature, sea level anomaly, and wind fields. Our results show that the timing of the CTW is influenced by variability in the equatorial region and along the southern African coast. Weaker equatorial easterlies from April to July delay the generation of the upwelling Kelvin wave, leading to a later arrival of the upwelling CTW. In contrast, the amplitude of the CTW is primarily influenced by variability in the eastern equatorial Atlantic and the central South Atlantic, where the South Atlantic Anticyclone is located. A cooling in the eastern equatorial Atlantic three to four months before the arrival of the CTW causes stronger zonal winds, ultimately leading to a stronger austral winter upwelling CTW. Our results suggest that the timing and amplitude of the upwelling CTW in the tAUS during austral winter are predictable on seasonal time scales.

**Supplementary Information:**

The online version contains supplementary material available at 10.1038/s41598-024-77917-2.

## Introduction

Angolan waters host a highly productive ecosystem of significant socio-economic importance: the tropical Angolan upwelling system (tAUS)^3–6^. Productivity in the tAUS follows a clear seasonal cycle as productivity peaks in austral winter in a narrow stripe along the Angolan coast^1,3,7^. In contrast to the Benguela upwelling region^8^, which is located poleward of the tAUS, the productivity in the tAUS is not controlled by local wind-driven upwelling^1^. Instead, the productivity maximum is associated with the passage of coastal trapped waves (CTWs) propagating along the African continent^1^.

**Fig. 1:**
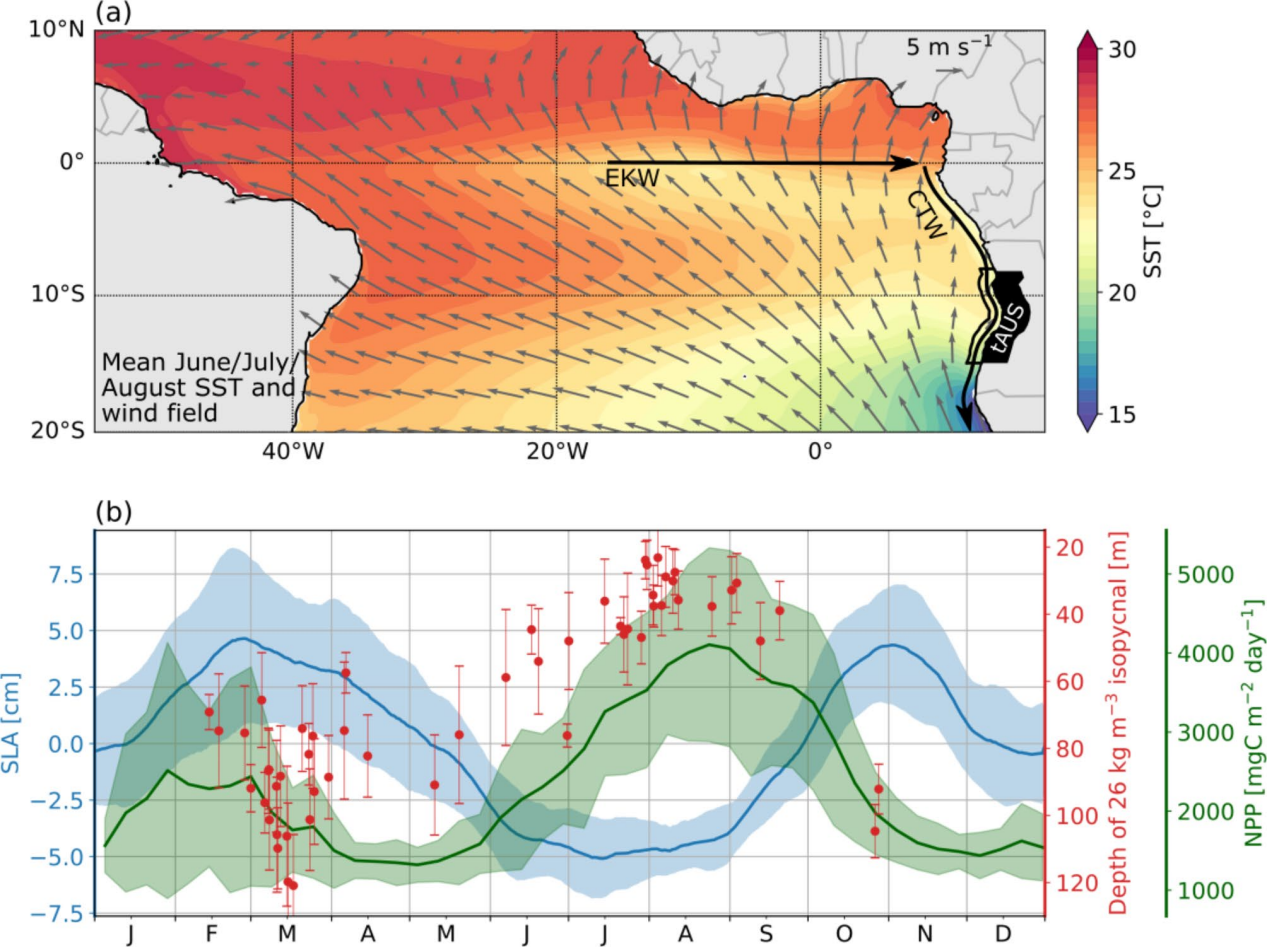
(**a**) Mean June/July/August sea surface temperature (SST) and wind fields. Black box marks the coastal strip of the tropical Angolan upwelling system (tAUS, 8°S–15°S, 1° distance to the coast). Black arrows indicate the propagation path of equatorial Kelvin waves (EKW) and coastal trapped waves (CTW). (**b**) Seasonal cycle of sea level anomaly (SLA), net primary production (NPP), and depth of the 26 kg m^−3^ isopycnal. The 26 kg m^−3^ isopycnal is a proxy for the nitracline^1^. The blue line shows the mean SLA for 1993–2022 derived from satellite data. The green line shows the mean NPP for 2003–2022 derived from satellite data. Shadings mark the standard deviations. Red dots indicate the depth of the 26 kg m^−3^ isopycnal averaged for individual cruises considering all hydrographic profiles taken between 250 and 1000 m water depth (see “Data and methods”). Red bars mark the standard deviation of the depth of the density surface. Data between 8°S and 15°S are used for all three variables. For both SLA and NPP, data within 1° off the coast are used. The map in (**a**) was created using the python packages matplotlib (version 3.8.4)^2^ and basemap (version 1.4.1, https://matplotlib.org/basemap/stable/).

CTWs observed at the continental slope off Angola are often remotely forced at the equator^9–11^. Zonal wind fluctuations at the equator excite equatorial Kelvin waves which propagate eastward. Upon reaching the eastern boundary part of their energy is transformed into westward propagating Rossby waves and part of it is transformed into poleward propagating CTWs^9,12,13^ (Fig. 1a). CTWs exhibit signals in sea level anomaly (SLA) where upwelling (downwelling) CTWs are associated with a depression (elevation) in sea level. SLA data from satellite retrievals reveal the passage of two upwelling and two downwelling CTWs throughout the year^1,3,6,10^. Körner et al.^1^ recently showed that the combined effect of the passage of upwelling CTWs and near-coastal mixing can explain the seasonal productivity maximum in the tAUS. They demonstrated that in austral winter CTWs of different vertical structure propagate along the Angolan shelf. First low-mode CTWs visible in SLA data arrive in the tAUS. Subsequently slower high-mode CTWs are observed, which displace the depth of the nitracline upward (Fig. 1b, the 26 kg m^−3^ isopycnal can be seen as a proxy for the nitracline). These high-mode CTWs are phase-locked to the low-mode CTWs visible in SLA^1^. The strength of the low-mode CTWs is correlated with the strength in primary production peaking more than a month after the SLA minimum. This delay together with the remote equatorial forcing of CTWs suggest a general predictability of the strength and timing of the productivity in the tAUS.

Equatorial dynamics influences the tAUS not only on seasonal time scales but also on intraseasonal and interannual time scales^11,14–16^. On interannual time scales, the strongest mode of variability in the tAUS are extreme warm and cold events, so-called Benguela Niños and Niñas which have been related to equatorially forced CTWs^11,15,17–19^. Benguela Niños and Niñas peak seasonally during the main downwelling season, between March and April^11,20,21^. Thus, their influence on the main productivity season in the tAUS is small. An exception was the 2021 Benguela Niño that peaked anomalously late in the year and strongly reduced the coastal productivity during austral winter^22,23^.

Both the equatorial Atlantic and the tAUS, as well as the connection between both systems, are subject to climate variability and change^24–28^. The sea surface temperature (SST) in the equatorial Atlantic warmed over the recent decades at the same time the variability of SST reduced^24,25^. Prigent et al.^25^ find a weakening in SST variability at the equator after the year 2000 associated with a weakening of the Bjerknes feedback and increased net surface heat flux damping. SST in the Angola Benguela area (ABA) warmed over the recent decades as well. Additionally, Roch et al.^29^ show that changes in temperature and salinity have led to intensification of stratification since 2006 in this region. Furthermore, variability of SST in the ABA reduced^26^. Possible reasons are associated with less influence of the equatorial remote forcing and a reduced thermocline feedback^26^. Model projections suggest a reduction of SST variability during the peak season of interannual variability in response to increasing greenhouse gas concentrations^28^.

In this study, we investigate the remote and local influences of tropical Atlantic variability on the timing and strength of the upwelling CTW in the tAUS in austral winter. The motivation of this study is the correlation between the amplitude of the upwelling CTW and the strength of the productivity peak in the tAUS^1^. Understanding the dynamical factors controlling the timing and amplitude of the upwelling CTW provides valuable insight into the variability of the productivity in the tAUS and could be useful for medium or long-term prediction of productivity in this region.

## Results

### Interannual variability of the austral winter SLA minimum in the tAUS

**Fig. 2:**
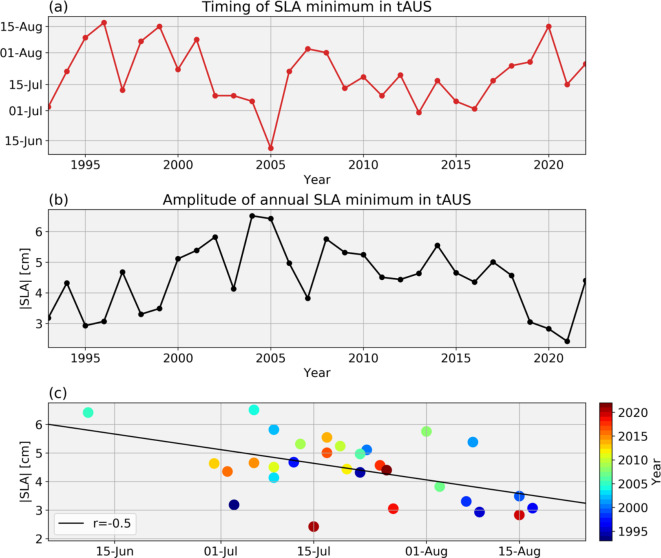
(**a**) Timing and (**b**) amplitude of the austral winter sea level anomaly (SLA) minimum in tAUS. SLA data was treated with a lowpass filter to find the timing of the annual minimum (cut-off frequency is 1/135 days^−1^). The amplitude of the SLA minimum is the absolute three-month mean SLA centered around its annual minimum. (**c**) Scatterplot of timing and amplitude of austral winter SLA minimum in tAUS as presented in (**a**),(**b**). Colors indicate the respective year of the data point. Linear regression line and the Pearson’s correlation coefficient, r, are also given.

SLA data have revealed an upwelling CTW propagating along the Angolan continental margin in austral winter^1,3,6,10^. In this study, we analyze the characteristics of the upwelling CTW as derived from its SLA signal. Specifically, we derive timing and amplitude of the SLA minimum that is associated with the upwelling CTW. Here, the amplitude of the SLA minimum describes the absolute peak of the annual SLA minimum, where higher amplitudes describe larger negative values of the SLA. Both timing and amplitude of the annual SLA minimum exhibit year-to-year variability (Fig. 2a,b).

Within the measurement period (1993–2022) the earliest occurrence date of the annual SLA minimum was on June 11th in 2005 while the latest occurrence date was on August 17th in 1996. On average the SLA minimum takes place on July 20th with a standard deviation of 15 days. The average amplitude of the SLA minimum is 4.5 cm with a standard deviation of 1.1 cm. The highest amplitude of the SLA minimum of 6.5 cm was recorded in 2004, while the lowest was 2.4 cm recorded in 2021. It should be noted that the amplitude of the SLA minimum also exhibits multidecadal variability (Fig. 2b) that are further discussed below.

The timing and amplitude of the austral winter SLA minimum are moderately correlated (Pearson correlation coefficient of − 0.5) (Fig. 2c). That means if the SLA minimum occurs earlier in the year the amplitude of the minimum tends to be higher. Nevertheless, the correlation between timing and amplitude of the SLA minimum is rather low, indicating that different mechanisms control their variability. Consequently, we investigate the influence of SLA, SST, and wind field on both, timing and amplitude of the CTW individually.

### Timing of the SLA minimum

**Fig. 3:**
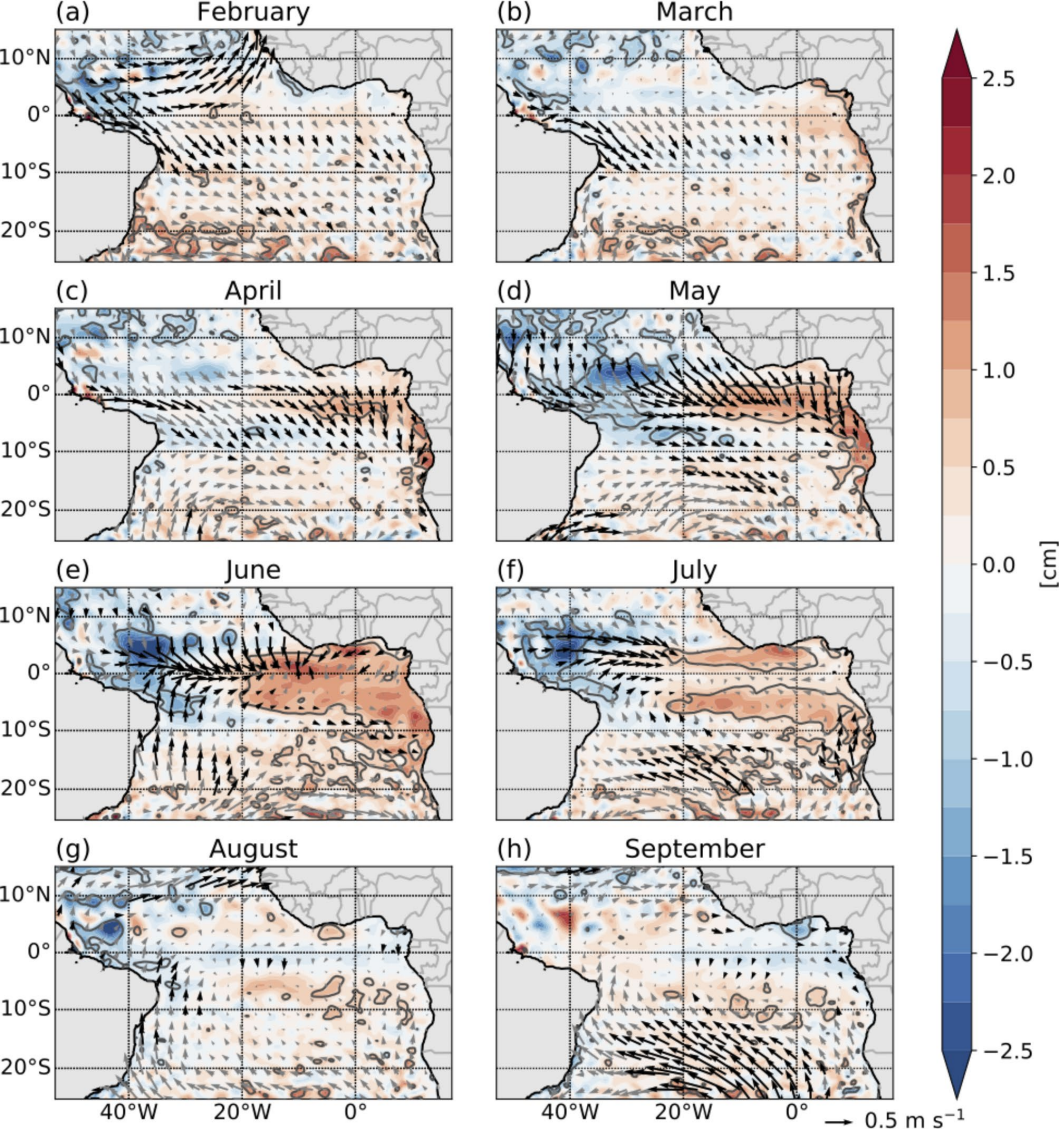
Maps showing the regression slopes of SLA (color) and wind (arrows) onto the timing of the SLA minimum in the tAUS as presented in Fig. 2a. The time series of timing of the SLA minimum is normalized prior to calculating the regressions (see methods). Regressions are calculated separately for the different months from February to September. Significant correlation (95% confidence level) between timing and SLA as well as timing and winds are marked by grey contour lines and black arrows, respectively. The regression slopes in the maps correspond to a late SLA minimum in the tAUS. The maps were created using the python packages matplotlib (version 3.8.4)^2^ and basemap (version 1.4.1, https://matplotlib.org/basemap/stable/).

Regression analyses were conducted to examine how the variability of wind, SLA, and SST in the tropical Atlantic is connected to the timing of the upwelling CTW in the tAUS during austral winter. For this, monthly anomalies of wind, SLA, and SST were regressed onto the timing of the SLA minimum. Monthly maps showing the regression slopes of SLA and wind for the period February to September are presented in Fig. 3, while the regression results for SST and wind are presented in Fig. S1. The derived regression slopes correspond to a late SLA minimum in the tAUS, indicative of a delayed arrival of the upwelling CTW.

The results of the regression analysis show large, coherent regions with statistically significant linear relationships between the wind, SLA and SST fields and the timing of the SLA minimum (Fig. 3 and Fig. S1). However, these coherent regions vary among different months.

North of the equator in February, northwesterly wind anomalies in the western part turning to westerly and southwesterly wind anomalies toward east, together indicative of weaker trade winds, are associated with a late arrival of the upwelling CTW (Fig. 3a). In this region, wind anomalies can excite Rossby waves, which propagate westward and can reflect into equatorial Kelvin waves^30–32^. Interestingly, and contrary to expectations, the Rossby wave signals are not visible in the SLA regression (Fig. 3a–c). However, the maps show significant correlations from February to April as indicated by higher SST in the western equatorial Atlantic (Fig. S1a–c). These SST signals could be connected to the presence of Rossby waves or due to changes in latent and sensible heat fluxes connected with the wind anomalies in this region.

The regression maps from April to July show statistically significant relationships between the timing of the SLA minimum and the equatorial wind and SLA field (Fig. 3c–f). At the equator, westerly wind anomalies, indicating weaker winds, are associated with the delayed arrival of the CTW. The spatial distribution of these anomalies evolves from the eastern equatorial Atlantic in April to the central and finally to the western equatorial Atlantic in July. Simultaneously, positive SLA signals in the eastern equatorial Atlantic in April transition into a dipole pattern with positive regression slopes in the east and negative regression slopes in the western equatorial Atlantic during May and June. In July, positive SLA north and south of the equator are linked to the late arrival of the upwelling CTW, while directly at the equator, no statistically significant correlation is visible. These patterns of regression of the timing of the SLA minimum in the tAUS onto the wind, and SLA fields show how weaker winds in different regions of the equatorial Atlantic during April to July lead to changes in the excitement of the equatorial Kelvin waves visible in the SLA pattern and ultimately to changes in the arrival time of the upwelling CTW in the tAUS. Noteworthy is the SLA pattern visible north and south of the equator in July. This pattern fits to the signal of a westward propagating downwelling Rossby wave which is forced by the reflection of a downwelling equatorial Kelvin wave at the eastern boundary prior to the late arrival of the upwelling equatorial Kelvin wave. In addition to the remote forcing of the timing of the upwelling CTW, the regression analysis also indicates local influences in July. Northward wind anomalies along the African coast, indicating stronger upwelling-favoring winds, contribute to a postponed SLA minimum, possibly by generating a late local upwelling signal (Fig. 3f).

In August and September, hardly any significant signals in the maps of the regression slopes of the wind and SLA onto the timing of the SLA minimum can be identified (Fig. 3g,h). One exception is the wind signal south of 10°S in September, suggesting that a late arrival of the upwelling CTW is followed by a stronger South Atlantic Anticyclone (SAA).

Similar to the SLA regression fields from April to July, the regression slopes of SST onto the timing of the SLA minimum show statistically significant correlation in the equatorial region as well (Fig. S1). In general, the regions showing a statistically significant correlation in SST are similar to the regions showing statistically significant correlation in SLA with positive SLA signals accompanied by a warming signal (cf. Fig. 3, Fig. S1). Positive SST signals in the eastern equatorial Atlantic in April indicate a late arrival of the upwelling CTW. This area of statistically significant correlation spreads in May and June covering nearly the whole equatorial Atlantic as well as large parts of the southeastern tropical Atlantic Ocean and the Gulf of Guinea. In July the regression pattern indicates a downwelling Rossby wave signal as statistically significant correlation with positive SST signals north and south of the equator are visible. This signal is persistent as it is still visible in August (Fig. S1g). In comparison to the SLA regression maps, the regression maps of SST show larger areas of statistically significant correlation, especially along the African coast.

Overall, the maps of regression slopes suggest that weakened equatorial easterly winds from April to July influence the generation of equatorial Kelvin waves, shaping SLA and SST patterns and resulting in a late arrival of the upwelling CTW in the tAUS. Furthermore, we find weakened trade winds north of the equator in February connected to a late arrival. A potential explanation for this signal is an induced warming of the western equatorial Atlantic.

### Strength of the SLA minimum

**Fig. 4:**
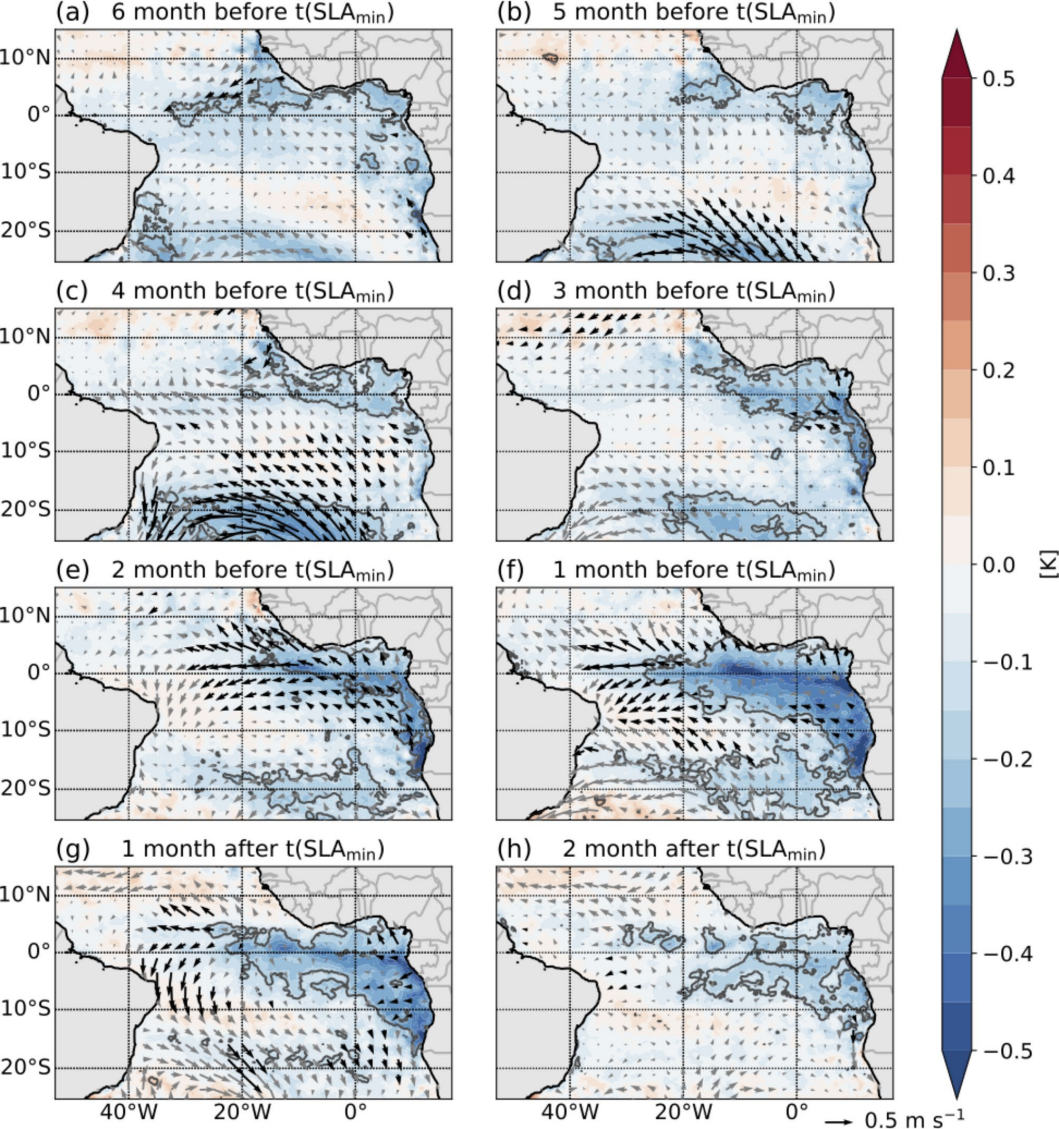
Maps showing the regression slopes of SST (colors) and winds (arrows) onto the amplitude of the SLA minimum in the tAUS presented in Fig. 2b. The time series of amplitude of the SLA minimum is normalized prior to calculating the regressions (see methods). Regressions are calculated for time periods relative to the timing of the annual SLA minimum (t(SLA_min_)) given in the titles (see methods). Significant correlation (95% confidence level) between the amplitude and SST and the amplitude and winds are marked by grey lines and black arrows. The regression slopes in the maps correspond to a higher amplitude of the SLA minimum in the tAUS. The maps were created using the python packages matplotlib (version 3.8.4)^2^ and basemap (version 1.4.1, https://matplotlib.org/basemap/stable/).

We regressed anomalies of wind, SLA, and SST onto the amplitude of the SLA minimum to understand the factors driving the variability in the strength of the upwelling CTW during austral winter. The anomalies of wind, SLA, and SST are not calculated as a function of calendar months but relative to the timing of the annual SLA minimum (see methods). This was done as we want to analyze the variability influencing the CTW characteristics relative to the arrival time of the wave. The maps, spanning from six months before to two months after the SLA minimum, for SST and wind, are presented in Fig. 4, with results for SLA and wind presented in Fig. S2. The derived regression slopes correspond to a higher amplitude of the SLA minimum, indicating a stronger upwelling CTW.

Similar to the maps of regression slopes for the timing of the SLA minimum, the results of the regression analysis show large coherent regions in which statistically significant linear relationships between the wind, SLA and SST fields and the amplitude of the SLA minimum (Fig. 4 and S2). However, the pattern and relationships vary from the ones discussed for the timing of the SLA minimum.

Five to four months before the SLA minimum, statistically significant correlation of the wind field in the South Atlantic onto the amplitude of the SLA minimum are visible (Fig. 4b,c). The maps suggest that a stronger SAA is linked to a higher amplitude of the SLA minimum and thus a stronger upwelling CTW. This signal is mirrored in SST, showing a statistically significant correlation with negative SST anomalies in the area of enhanced wind anomalies. Similar patterns are observed in SLA, with a patchier negative SLA signal in the same region (Fig. S2a,b). The fact that the same region shows up in all three variables suggests that there is indeed a connection between the SAA strength and the amplitude of the upwelling CTW five to four months later. Additionally, at the same time negative SST signals in the Gulf of Guinea and the eastern equatorial Atlantic that are statistically significant, correspond to a higher amplitude of the SLA minimum, with no corresponding signal in wind and SLA in this region. This raises the question of the origin of the cooling in the eastern equatorial Atlantic. One possible explanation is that the cooling in this area is connected to a stronger SAA. A stronger SAA could influence the distribution of clouds over the tropical Atlantic Ocean inducing the cooling by reduced short wave radiation. To check this hypothesis, we conducted regression analyses of the total cloud cover on the amplitude of the SLA minimum (Fig. S3). The results indicate that four to three months before the SLA minimum higher cloud cover over the eastern equatorial Atlantic is connected to a higher amplitude of the SLA minimum. Note that only a small area of this pattern shows a significant correlation questioning the robustness of this result. Nevertheless, this theory would offer an explanation of the connection between the SAA strength, the cooling of the eastern tropical Atlantic, and the amplitude of the SLA minimum five to four months later.

Three months before the SLA minimum, the statistically significant correlation with the wind field of the SAA vanishes (Fig. 4d). However, the SST and SLA signals in the region are persistent (Fig. 4d and Fig. S2d). Additionally, the SST signal in the eastern equatorial Atlantic remains as well.

A statistically significant correlation between the amplitude of the SLA minimum and the equatorial wind field is visible 2 months before the SLA minimum (Fig. 4e). Easterly wind anomalies in the eastern and central equatorial Atlantic, indicating stronger equatorial easterlies, are associated with a higher amplitude of the SLA minimum. These easterly wind anomalies are likely a response of the wind field to the lower SST visible one months earlier (Fig. 4d). The statistically significant negative SST signals are also visible 2 months before the SLA minimum (Fig. 4e). The SST signal becomes stronger, and the area of this signal expands covering the eastern equatorial Atlantic, Gulf of Guinea, and extends along the southern African coast up to the Angola-Benguela Frontal Zone (ABFZ). One month before the SLA minimum, the area of statistically significant correlation of the wind field shifts westward (Fig. 4f). At the same time the area of the statistically significant negative SST signal expands, now covering the equatorial region east of 25°W. Similar signals as the ones for SST are observable for SLA (Fig. S2e,f).

One month after the SLA minimum the regression maps still indicate colder-than-usual SSTs in the equatorial region and along the Angolan coast connected to a stronger upwelling CTW, although the signal is weaker (Fig. 4g). Meanwhile, a statistically significant stronger wind signal in the western equatorial Atlantic is visible. Two months after the SLA minimum, no statistically significant correlation is observed in the regression pattern of the wind, however, in the eastern equatorial Atlantic a negative SST signal remains.

The regression maps of SST, SLA, and wind onto the amplitude of the SLA minimum unveil distinct regions influencing the strength of the upwelling CTW. An influence of the strength of the SAA is evident five to four months before the SLA minimum, where a stronger SAA is connected a stronger upwelling CTW. The strengthening of the SAA may lead to a shift in cloud cover leading to an atmospheric-driven cooling in the eastern equatorial Atlantic. However, more work is needed to understand the possible connection between the SAA and cooling in the eastern equatorial Atlantic. Two months before the SLA minimum the equatorial wind field reacts to the stronger zonal SST gradient, with the maps illustrating that a stronger wind field in the eastern and central equatorial Atlantic leads to enhancement of the negative SST anomalies and negative SLA and subsequently a stronger upwelling CTW. This regression analysis reveals the remote influences of both the SAA strength and equatorial wave dynamics on the amplitude of the upwelling CTW.

### Temporal evolution of the equatorial connection

Prigent et al.^26^ showed that the respective role of remote equatorial to local forcing of SST anomalies in the ABA changes over time. Here, we analyze the amplitude of the SLA minimum at the equator and in the tAUS to assess changes in the relationship between both regions. A high co-variation is expected if the strength of SLA minimum in the tAUS is strongly related to equatorial dynamics.

**Fig. 5:**
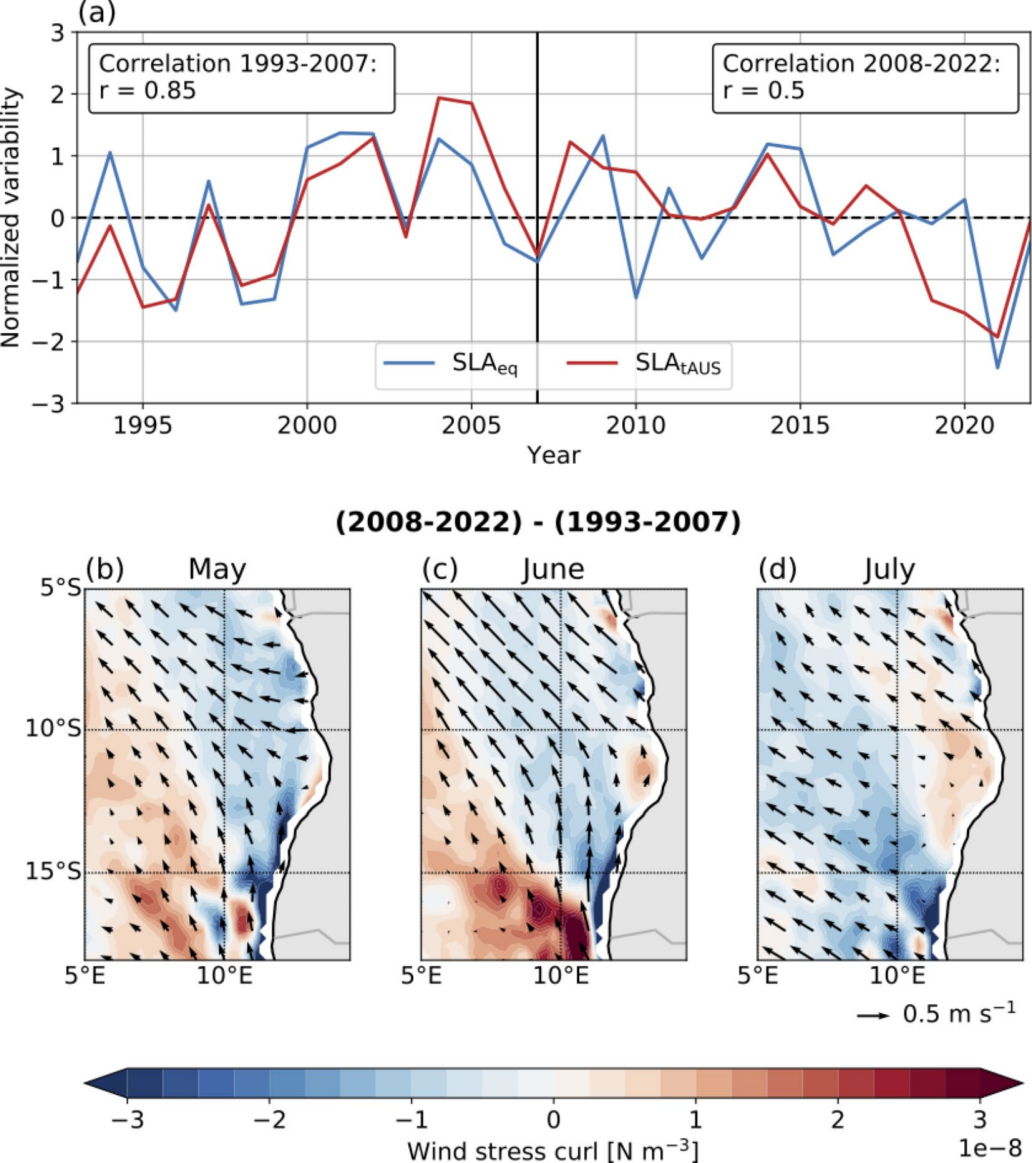
(**a**) Amplitude of SLA minimum in austral winter in the tAUS (red; 8°S–15°S, 1° distance to the coast) and at the equator (blue; 20°W–0°, 1°S–1°N). Both time series are normalized by their respective standard deviation. The Pearson correlation coefficient, r, between both time series is given for the two different time periods. (**b**–**d**) Difference of the wind field (arrows) and the wind stress curl (colors) between 2008–2022 and 1993–2007 for the months May, June and July. The maps were created using the python packages matplotlib (version 3.8.4)^2^ and basemap (version 1.4.1, https://matplotlib.org/basemap/stable/).

Figure 5a shows the time series of the amplitude of the austral winter SLA minimum at the equator (20°W–0°E; 1°N–1°S) and in the tAUS (8°S–15°S, 1° distance to the coast). It becomes evident that the SLA minima of both regions undergo long-term variability. Moreover, a distinct shift in the relationship between the SLA minima becomes apparent during the period 1993–2022. From 1993 to 2007, the amplitude of the SLA minimum at the equator and in the tAUS exhibits a close connection, evident through a Pearson correlation coefficient of 0.85 (significant at a 95% confidence level). After 2007 the correlation is much weaker (Pearson correlation coefficient of 0.5, not significant at 95% confidence level) indicating a diminished connection between the two regions. Concurrently, there is a reduction in the variability of the amplitude of the SLA minimum in both areas. In the tAUS, the standard deviation decreases by 18%, declining from 1.16 cm (1993–2007) to 0.95 cm (2008–2022). At the equator, the reduction is somewhat weaker as the standard deviation decreases by 10%, shifting from 1.54 cm (1993–2007) to 1.38 cm (2008–2022). Thus, over the measurement period, the variability in each region decreases, accompanied by a decline in the correlation between the two regions. It is worth mentioning that the last two years of the time series exhibit strong co-variability again.

We want to analyze possible reasons for this changing behavior. Note that we abstain from calculating regressions for both time periods separately as this would mean regression on a very short time series. Especially in the second period little variability in the time series would make the analyses less robust. Instead, we focus on examining mean state changes in the wind fields between the two time periods. Figure 5b–d shows that for 2008–2022 the local wind fields show stronger alongshore wind in May–July compared to 1993–2007. Especially in May and June differences are visible. The changes in wind are associated with negative wind stress curl anomalies (i.e., upwelling-favoring anomalies) along the coast during these months. These results suggest a strengthening of local forcing in the second period, potentially weakening the connection to the equator.

## Discussion

The austral winter upwelling season in the tAUS is determined by the arrival of the upwelling CTW propagating along the equatorial and coastal waveguides. In this study, we investigate the interannual variability of the timing and amplitude of the austral winter upwelling CTW in the tAUS, along with their potential controlling parameters. The timing and amplitude of the SLA minimum, used as a proxy for the CTW in tAUS, show a weak correlation between the two variables. This suggests that the forcing mechanisms of the upwelling CTW may differ.

The timing of the SLA minimum (i.e., the timing of the upwelling CTW) seems to be mainly influenced by equatorial variability from April to July. In these months weaker winds, warmer SSTs and a dipole structure in SLA with negative signals in the west are associated with a late SLA minimum. The patterns fit well with how wind anomalies in the equatorial region alter the seasonal excitement of equatorial Kelvin waves, shaping thus SST and SLA pattern and affecting the arrival of the upwelling CTW in the tAUS. Furthermore, there is an indication that the strength of trade winds north of the equator in February plays a role in the timing of the SLA minimum. This signal in the wind field is connected to a warming of the western equatorial Atlantic, which could be linked to the presence of Rossby waves or changes in latent and sensible heat fluxes.

The amplitude of the SLA minimum (i.e., the strength of the upwelling CTW) appears to be linked to the strength of the SAA five to four months prior to the SLA minimum, with a stronger SAA associated with a higher amplitude of the SLA minimum. The strengthening of the SAA may influence the cloud cover over the eastern equatorial Atlantic, leading to a cooling in this region. However, the connection between the strength of the SAA and the cooling of the eastern equatorial Atlantic is not robust, requiring further investigation to understand how these signals are connected. The wind field in the eastern equatorial Atlantic responds to the cooling with eastward anomalies two to one month before the SLA minimum. These eastward wind anomalies in turn likely affect the strength of the equatorial Kelvin wave, leading to negative SLA and negative SST signals at the equator and subsequently to a higher amplitude of the SLA minimum in the tAUS.

**Fig. 6:**
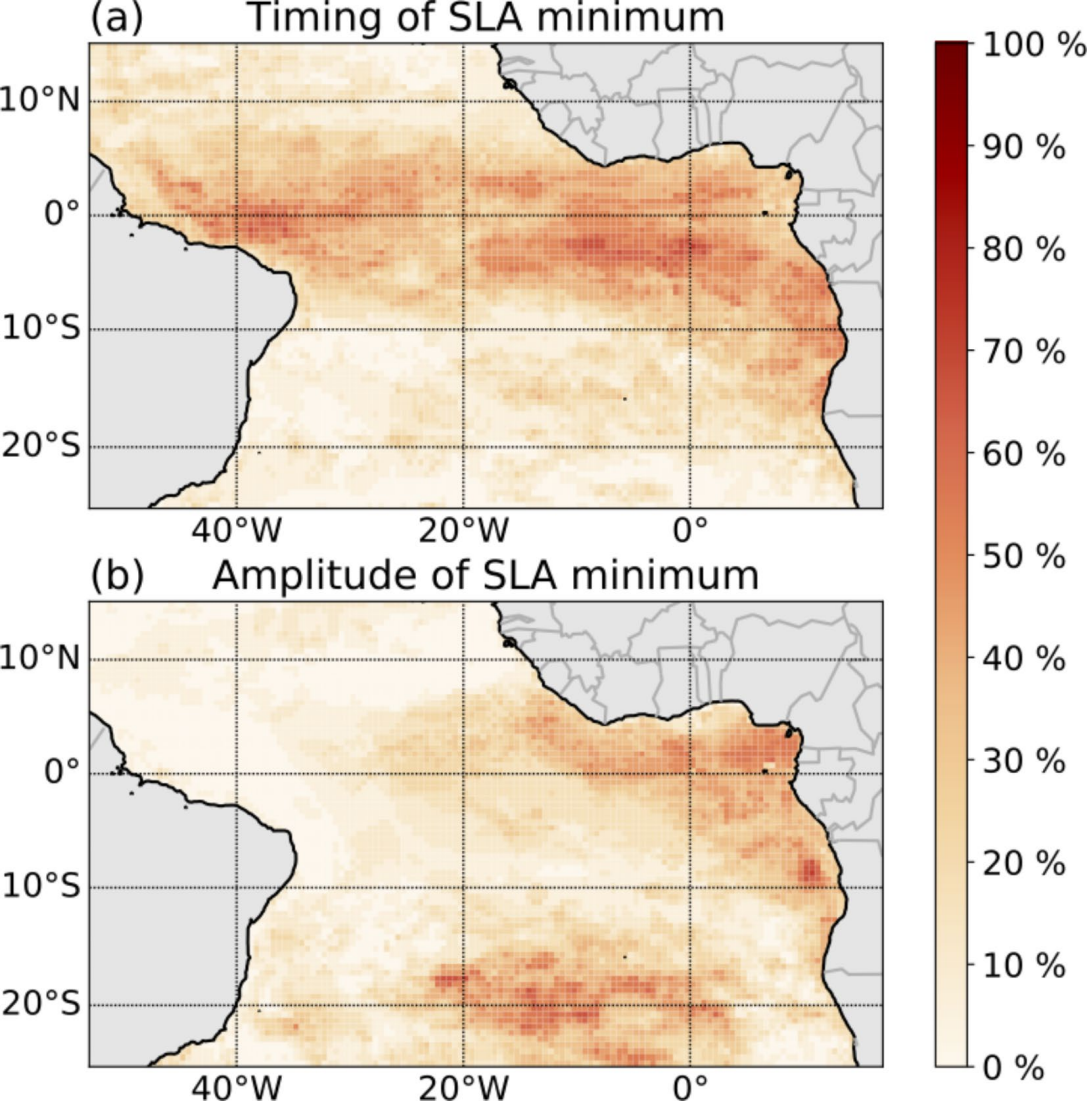
Heat map of significant correlation (95% confidence level) of the SST, SLA and wind field onto (**a**) the timing of the SLA minimum and (**b**) the amplitude of the SLA minimum. Pixel showing 100% corresponds to a location with a significant correlation in all three variables for the whole time period. The heat map is calculated for the time period of (**a**) February–July and (**b**) six–one month before the SLA minimum. The maps were created using the python packages matplotlib (version 3.8.4)^2^ and basemap (version 1.4.1, https://matplotlib.org/basemap/stable/).

These regression analyses suggest that the timing and amplitude of the austral winter upwelling CTW in tAUS are influenced by variability in different regions. We calculate heat maps to identify regions with significant correlations of SST, SLA, and wind with the timing and amplitude of the SLA minimum, respectively (Fig. 6). Figure 6a clearly shows that the timing of the SLA minimum is primarily influenced by variability in the equatorial region and along the Southwest African coast north of the ABFZ. The amplitude of the SLA minimum is influenced by variability in the eastern equatorial Atlantic and the central South Atlantic, a region characterizing the strength of the SAA (Fig. 6b).

Our results further show that the connection between the variability of the amplitude of the SLA minimum at the equator and in the tAUS has changed. Between 1993 and 2007 the amplitude of the SLA minimum in the tAUS and at the equator exhibited high correlation, while between 2007 and 2022 the correlation weakened and the variability in both regions declined. Prigent et al.^26^ investigated the connection between equatorial SST variability and SST variability in the ABA focusing on the peak season of interannual variability (March–May). They found that after the year 2000, the influence of equatorial forcing on SST variability of ABA variability weakened while local atmospheric influences became more important.

The question arises of the origin of the changes in the co-variability between the two regions. Historic CMIP6 model runs show a decline in tropical Atlantic SST variability since the 1970s^33^. Furthermore, model projections suggest a reduction of SST variability in the ABA in response to increasing greenhouse gas concentrations^28^. Climate projections further suggest a weakening of Atlantic Niño variability, attributed to a decoupling of subsurface and surface processes, i.e. a reduced thermocline feedback^34,35^. However, high decadal to multidecadal variability in the tropical Atlantic variability might influence the results as well^33,36,37^. Additionally, the influence of the El Niño/Southern Oscillation (ENSO) has experienced a reduction in variability in recent decades as well^38–40^ which is of interest for the Atlantic Ocean as ENSO influences the tropical Atlantic variability^41,42^. Thus, the existing studies show a complex picture of long-term variability and response to global warming with an overall expectation of reduced variability in the future^28^. Note that in the last few years variability at the equator and in the ABA picked up again, with major warm events recorded in 2019 and 2021^22,23,30,32^. This underlines that further work is needed to understand the future of tropical Atlantic variability. Our study underlines that this is important not only in the context of SST variability and extreme events but also for the year-to-year variability of the upwelling season and associated primary productivity in the tAUS.

The amplitude of SLA minimum undergoes multidecadal variability (Fig. 2c). Note that in this study we remove the linear trend in SLA. The linear trend of the amplitude of the SLA minimum over the period of 1993–2022 is 3.6 mm year^−1^, the acceleration is 0.2 mm year^−2^ (determined via a second order polynomial fit). Globally, sea level rises as a response to climate warming which is accelerating since the 1960s^43^. Global estimates shows a linear sea level rise of 3.3 ± 0.3 mm year^−1^ and an acceleration of 0.12 ± 0.05 mm year^−2^ over the period 1993–2021^44^. This suggests that part of the multidecadal variability in amplitude of the SLA minimum can potentially be attributed to global warming. Especially, as Dangendorf et al.^43^ showed that the acceleration of sea level rise in the South Atlantic is higher than the global average. However, climate variability might also contribute to the multidecadal variability. Here, the changes in the SAA and its influence on the tAUS could play a role. Averaging wind speed anomalies between 20°W–0°E and 30°S–20°S 5 months before the SLA minimum show similar multidecadal variability as well (not shown). This underlines the possible influence of the SAA on the variability of the amplitude of the SLA minimum.

The present study gives valuable insights how variability in different areas of the tropical and central South Atlantic influences the timing and amplitude of the austral winter upwelling CTW in tAUS. Understanding what drives the variability of the upwelling CTW is of great interest to understand the variability of primary production, as the amplitude of the CTW is related to the strength of the productivity peak in the tAUS in austral winter^1^. Maps of regression slopes of net primary production (NPP) onto the timing and the amplitude of the SLA minimum confirm this relationship (Figs. S4 and S5). Note that this analysis covers a shorter period as NPP data is only available since 2002. Furthermore, variability in the position and strength of the Congo River plume leads to strong signals in the plume region. Nevertheless, the maps show, connected with a late arrival of the CTW, less NPP in Angolan waters in June and July. Additionally, connected with a stronger CTW, higher NPP are found in Angolan waters one month before until 3 months after the SLA minimum. Summarizing, this study thus offers the groundwork for future attempts to predict the timing and strength of the CTW and thus productivity in the tAUS during the main upwelling season.

The tAUS is not the only tropical upwelling system where productivity is influenced by CTWs. In the Peruvian upwelling system, equatorially forced intraseasonal CTWs can trigger phytoplankton blooms that are largely responsible for intraseasonal chlorophyll variability^45^. Similar to the tAUS, higher-mode CTWs play a crucial role in this process^45^. This raises the question of whether similar predictive mechanism, possibly on different time scales, might exist in other tropical upwelling system as well.

## Data and methods

### Data

To investigate the amplitude and timing of the austral winter upwelling CTW we make use of different satellite data products.

The SLA data used in this study are from Copernicus (10.48670/moi-00148). The daily SLA fields are available with a horizontal resolution of a 0.25° $\times$ 0.25° grid since 1993.

The SST fields used in this study are from the OSTIA product^46^. The OSTIA product uses satellite data as well as in situ measurements to provide global, daily, gap-filled SST fields. The data are available on a 0.05° $\times$ 0.05° grid from 1981 onward. Note that there are two versions of the OSTIA product available from Copernicus. One spans the time period from September 1981 to May 2022 (10.48670/moi-00168). The other spans the time period from December 2006 to the present (10.48670/moi-00165). For this study, we use data from the first version from January 1993–May 2022 and data from the second version from June 2022–December 2022. To make the calculation more efficient, we subsampled the SST fields onto a grid with a horizontal resolution of 0.25° $\times$ 0.25° before perfomring the regression analyses.

The wind speed and cloud cover data used in this study are from the ERA5 reanalysis product^47^. The zonal and meridional components of wind speed 10 m above the earth’s surface and the cloud cover are available on a horizontal grid with a resolution of 0.25° $\times$ 0.25°. The hourly data are available from 1940 onward. We used daily averages in this study.

The net primary production (NPP) data are from the Oregon State University (http://sites.science.oregonstate.edu/ocean.productivity/index.php). NPP uses the Eppley Vertically Generalized Production Model. It is based on chlorophyll concentration, SST, and photosynthetically available radiation from MODIS. The NPP fields are gap-free and have a horizontal resolution of 1/6° $\times$ 1/6°. The data is available from July 2002 onward.

The analysis in this study uses data from January 1993 to December 2022 as data from most products are available for this time period. For the regression analysis of NPP on timing and amplitude of the SLA minimum (Figs. S4 and S5), we use data from January 2003–December 2022.

The seasonal cycle of the depth of the 26 kg m^−3^ isopycnal presented in Fig. 1b is derived using data from research cruises conducted from 1995 to 2022 in the tAUS. For more information on this dataset the reader is referred to Körner et al.^1^.

### Determining timing and amplitude of SLA minimum

The timing and amplitude of the annual minimum in SLA are calculated considering the time series of SLA averaged in the tAUS. To derive the time series of SLA in the tAUS we first remove the linear trend in SLA data at each grid point. Subsequently, we average SLA over the area of the tAUS considered in this study (8°S–15°S, 1° distance to the coast). Finally, high-frequency variability is eliminated by applying a cosine-Lanczos filter as formulated by Thomson and Emery^48^. Here, a cutoff frequency of 1/135 days^− 1^ was chosen to remove the effect of intraseasonal CTWs with periods of 90 and 120 days that are commonly observed in the tAUS^16^. To determine the amplitude of the SLA minimum, we average 90 days of the unfiltered SLA time series centered around its annual minimum.

### Regression analysis

To understand how variability in the tropical Atlantic influences the timing and amplitude of the upwelling CTW in the tAUS, we conduct regression analyses. We regress various predictors (e.g., wind, SLA, SST) onto the explanatory variables timing and amplitude of the annual SLA minimum in the tAUS. The quality of the regression and its statistical significance is evaluated using the Pearson correlation coefficient and its p-value. In this section, we first outline our general method, followed by a detailed example to illustrate its application.

Before calculating the regressions, the explanatory variables were detrended and normalized by their respective standard deviation. Thus, the time series of the timing and amplitude of the SLA minimum are unitless and the regression slopes have the unit of the predictor variable. Consequently, the regression slopes relate to the standardized explanatory time series and do not represent absolute changes of the explanatory variables.

For the regression analysis on the timing of the SLA minimum, monthly anomalies of SLA, SST, wind and NPP were considered as predictors. Individual linear regressions of these fields were calculated at each grid point. Prior to calculating the regressions, the predictors were detrended at each grid point separately. Here, the mean and the trend were calculated for the time series at this grid point of the respective month. Thus, the trend of the respective month is removed and not the trend of the full time series. The predictors were regressed onto the normalized time series of the timing of the SLA minimum. The maps in Fig. 3, Figs. S1 and S4 thus depict the regression slopes leading to a late SLA minimum in the tAUS.

The regression analysis on the amplitude of the annual SLA minimum is calculated for the predictors SLA, SST, wind, NPP, and cloud cover. The regressions are calculated for the anomalies relative to the timing of the annual SLA minimum. For that, the daily anomalies of the SLA, SST and wind fields are calculated by removing the daily climatology. We then map the anomalies as a function of the lag in days relative to the annual SLA minimum as presented in Fig. 2a. The anomalies are then averaged in bins with a bin size of 30 days. Thus, the resulting fields depict the SLA, SST, wind, NPP, and cloud cover anomalies relative to the timing of the annual SLA minimum. Before calculating the regression, the predictor time series at each grid point were detrended. The mean and the trend are calculated for the time series at this grid point over the respective time period. The predictors were regressed onto the normalized time series of the amplitude of the SLA minimum. The maps in Fig. 4, Figs. S2, S3 and S5 thus show the regression slopes leading to a higher amplitude of the SLA minimum in the tAUS.

After calculating the regression slopes, we evaluate the statistical significance of the regression using the same predictor and explanatory time series as used for the calculation of the regression slopes. The significance is evaluated by calculating the Pearson correlation coefficient and testing it against the null hypothesis, which states that the underlying distributions of the samples are uncorrelated and normally distributed. The p-value expresses the probability of observing zero correlation. In this study, we use a threshold of *p* < 0.05, corresponding to a 95% confidence level, to classify statistically significant correlations.

To illustrate our methodology in detail, we provide a specific example using SST in the month of May at 0°N; 0°E as a predictor for the timing of the SLA minimum (Fig. S6). In a first step, the mean of the monthly SST time series for May (Fig. S6a) is removed and the time series is detrended (Fig. S6b). This time series is then regressed onto the normalized timing of the SLA minimum in the tAUS (Fig. S6c). The slope of the regression ($m={0.4}^{\circ }C$) indicated that years with higher SST at 0°N, 0°E in May are associated with a late SLA minimum in the tAUS. The Pearson correlation coefficient ($r=0.69$) and its p-value ($p=2\times {10}^{-5}$) are calculated to measure the strength and significance of the relationship between the two variables. The p-value indicates a statistically significant correlation for the example presented here.

## Electronic Supplementary Material

Below is the link to the electronic supplementary material.

Supplementary Material 1

## Data Availability

Publicly available datasets were used for this study. The sea level anomaly data can be accessed via Copernicus server (10.48670/moi-00148). The sea surface temperature data are from the OISTA product (10.48670/moi-00168, 10.48670/moi-00165). The wind and cloud cover data are from ERA5 (10.24381/cds.adbb2d47). The hydrographic cruise data used in this study are available at 10.1594/PANGAEA.886492 and https://zenodo.org/records/10062790. The code to reproduce the analyses and figures of this study is available at https://doi.org/10.5281/zenodo.13765786.
