# Supplementary material for: Strength and timing of austral winter Angolan coastal upwelling
Source: Sci Rep. 2024 Nov 9;14:27400. doi: 10.1038/s41598-024-77917-2 (PMC11550842; doi:10.1038/s41598-024-77917-2)
Supplement: Supplementary file 1 — Supplementary Material 1 [file 41598_2024_77917_MOESM1_ESM.pdf]

Supplementary Materials for

Strength and timing of austral winter Angolan coastal upwelling

Mareike Körner *et al.*

*Corresponding author. Email: mareike.koerner@oregonstate.edu

This PDF file includes:

Figs. S1 to S6

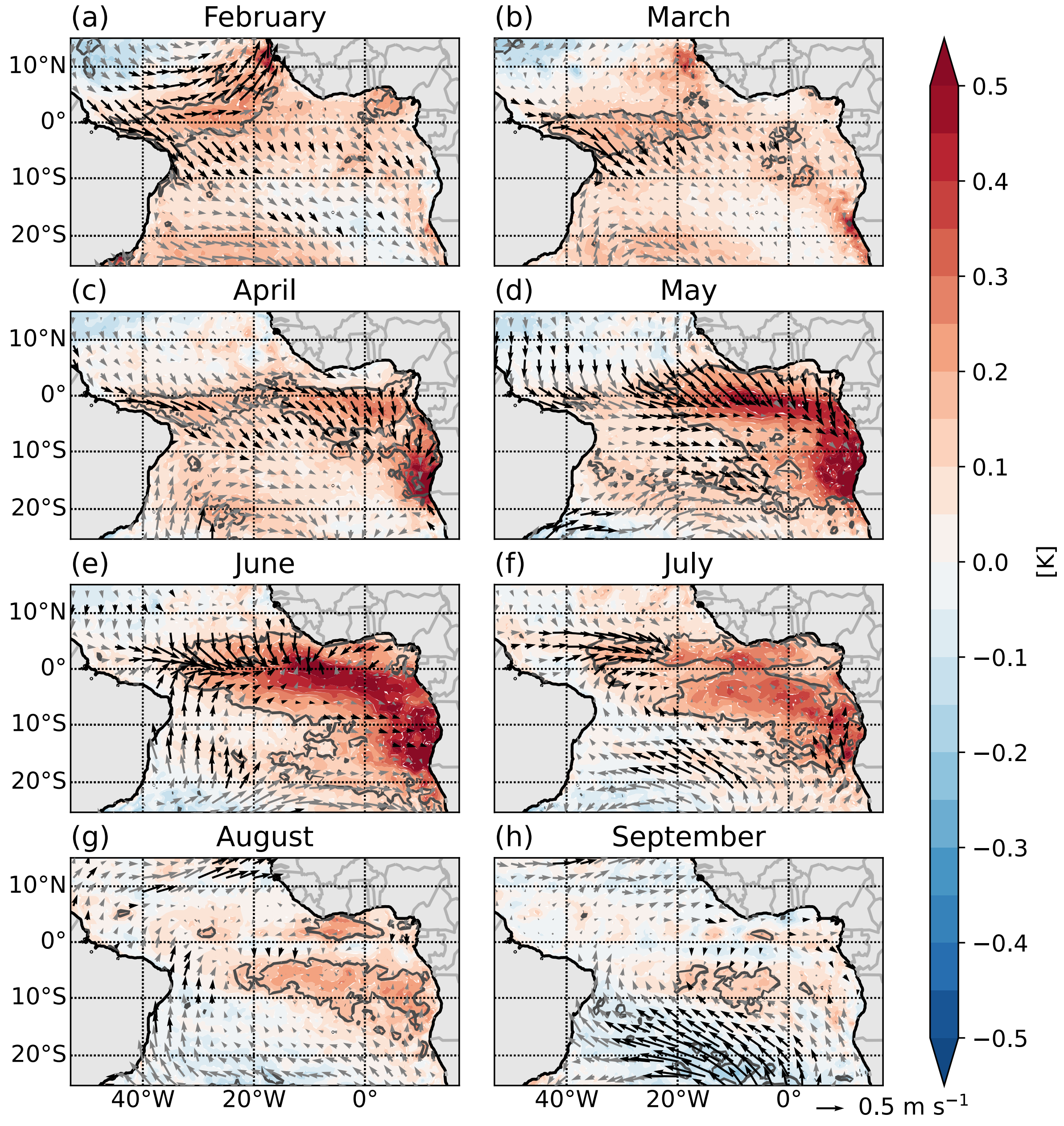

Figure S1: Maps showing the regression slopes of SST (color) and wind (arrows) onto the timing of the SLA minimum in the tAUS as presented in Fig. 2a. The time series of timing of the SLA minimum is normalized prior to calculating the regressions (see methods). Regressions are calculated separately for the different months from February to September. Significant correlation (95% confidence level) between the timing and SLA and the timing and winds are marked by grey lines and black arrows. The regression slopes in the maps correspond to a late SLA minimum in the tAUS. The maps were created using the python packages matplotlib (version 3.8.4)[2] and basemap (version 1.4.1, https://matplotlib.org/basemap/stable/).

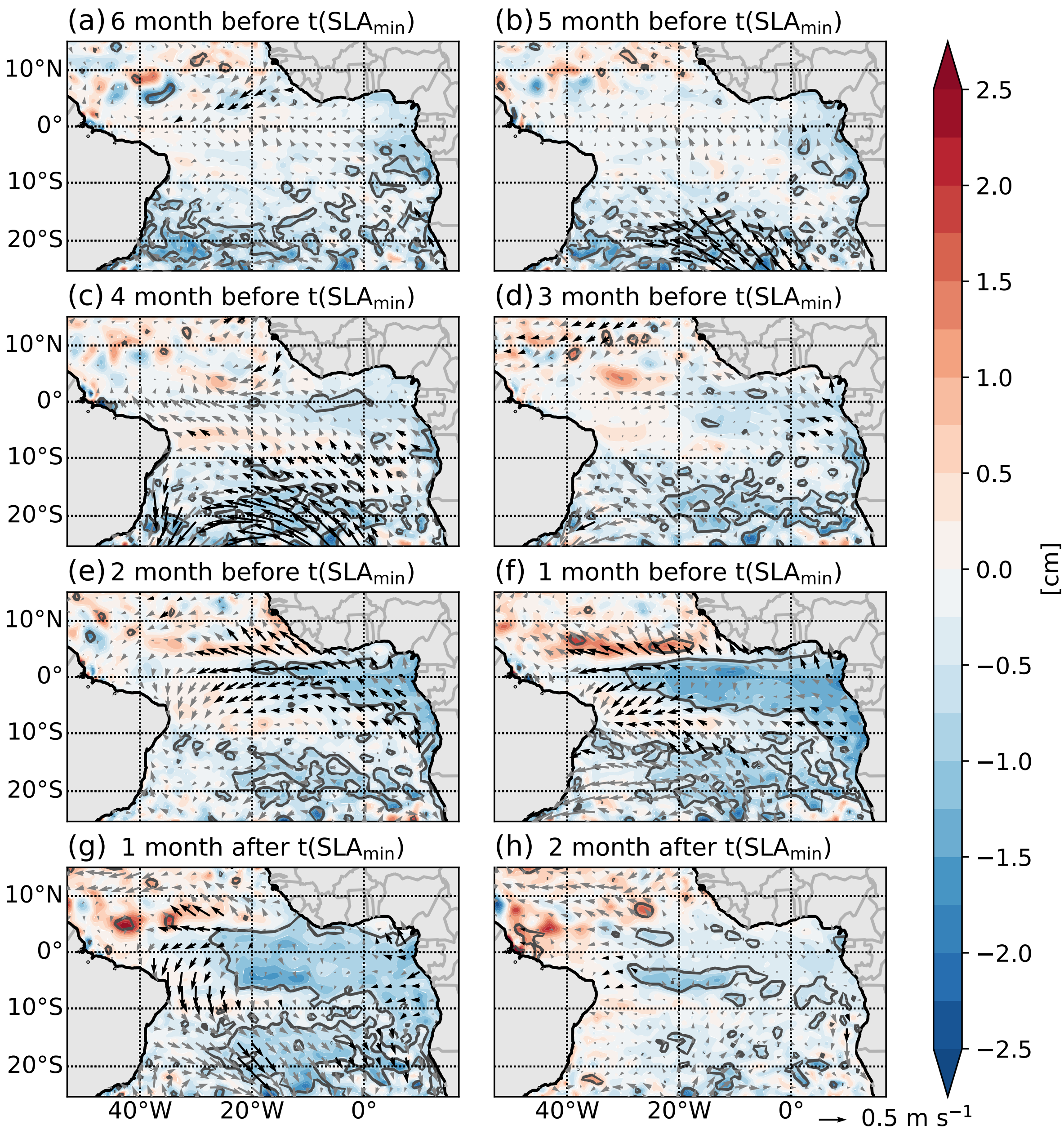

Figure S2: Maps showing the regression slopes of SLA (colors) and winds (arrows) onto the amplitude of the SLA minimum in the tAUS presented in Fig. 2b. The time series of amplitude of the SLA minimum is normalized prior to calculating the regressions (see methods). Regressions are calculated for time periods relative to the timing of the annual SLA minimum given in the titles (see methods). Significant correlations (95% confidence level) between the amplitude and SLA and the amplitude and winds are marked by grey lines and black arrows. The regression slopes in the maps correspond to a higher amplitude of the SLA minimum in the tAUS. The maps were created using the python packages matplotlib (version 3.8.4)[2] and basemap (version 1.4.1, https://matplotlib.org/basemap/stable/).

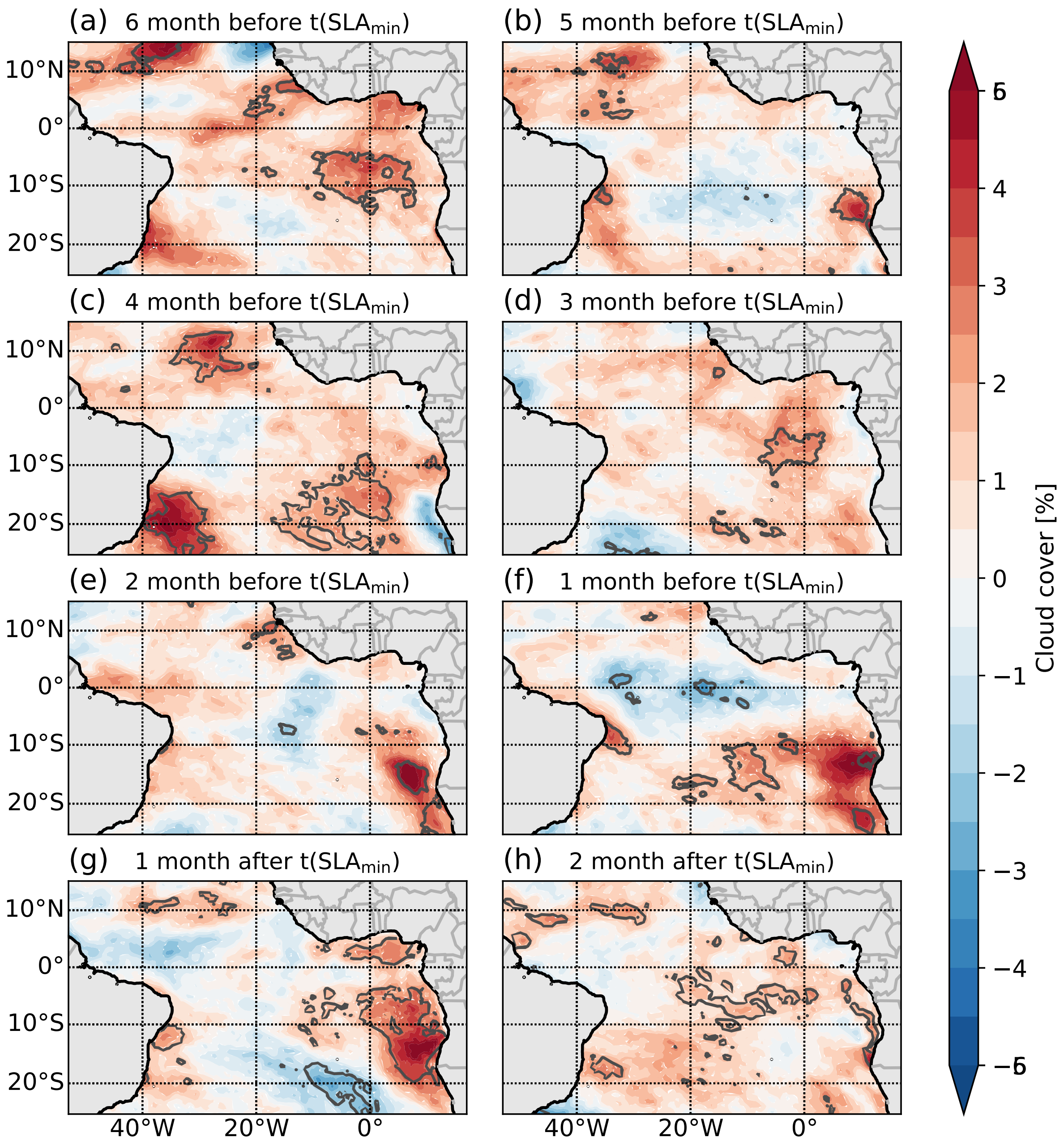

Figure S3: Maps showing the regression slopes of total cloud cover (colors) onto the amplitude of the SLA minimum in the tAUS presented in Fig. 2b. The time series of amplitude of the SLA minimum is normalized prior to calculating the regressions (see methods). Regressions are calculated for time periods relative to the timing of the annual SLA minimum given in the titles (see methods). Significant correlations (95% confidence level) between the amplitude of the SLA minimum and the total cloud cover are marked by grey lines. The regression slopes in the maps correspond to a higher amplitude of the SLA minimum in the tAUS. The maps were created using the python packages matplotlib (version 3.8.4)[2] and basemap (version 1.4.1, https://matplotlib.org/basemap/stable/).

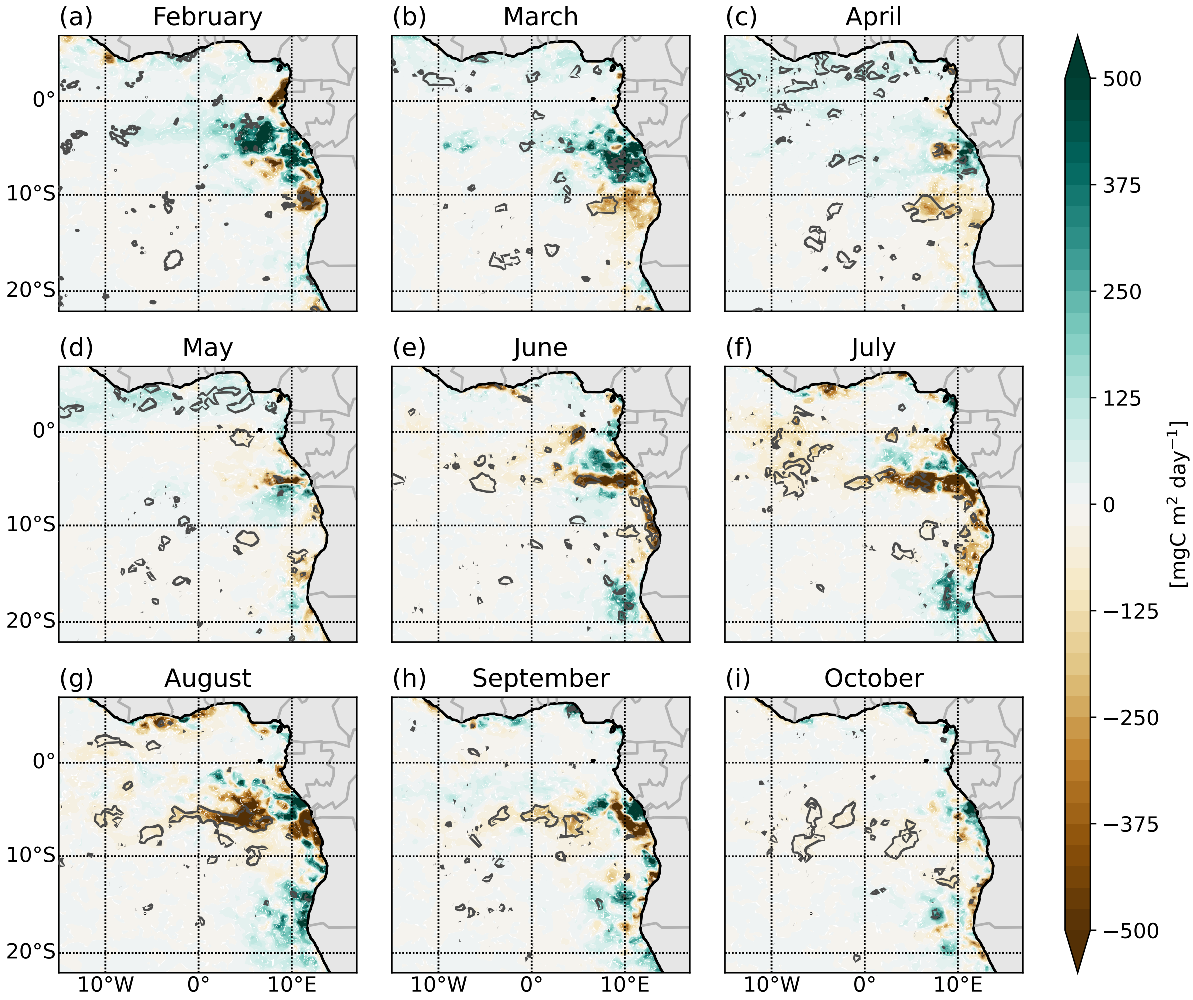

Figure S4: Maps showing the regression slopes of NPP onto the timing of the SLA minimum in the tAUS presented in Fig. 2a for the period 2003-2022. The time series of timing of the SLA minimum is normalized prior to calculating the regressions (see methods). Significant correlation (95% confidence level) is marked by grey lines. The regression slopes in the maps correspond to a late SLA minimum in the tAUS. The maps were created using the python packages matplotlib (version 3.8.4)[2] and basemap (version 1.4.1, https://matplotlib.org/basemap/stable/).

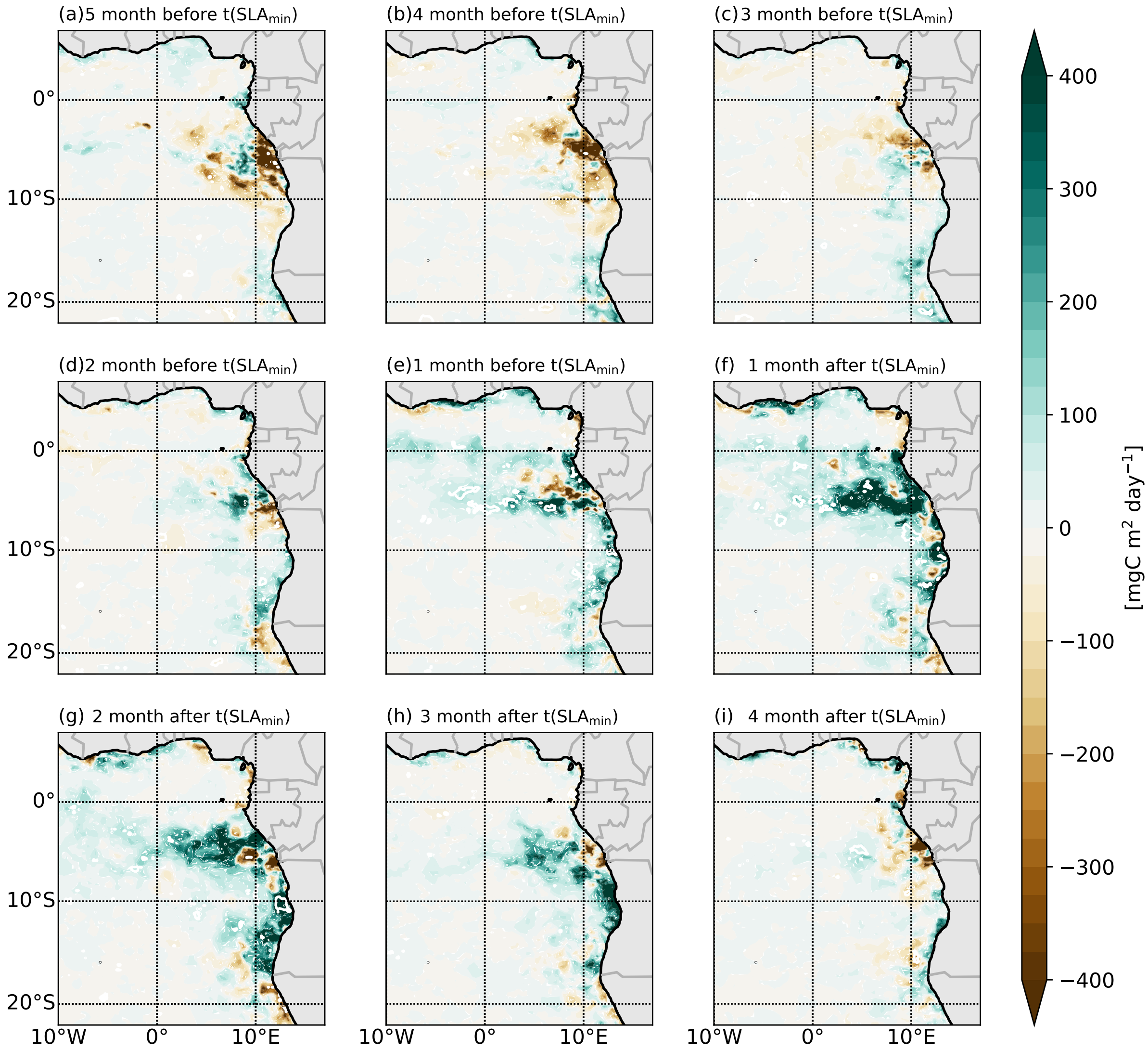

Figure S5: Maps showing the regression slopes of NPP onto the amplitude of the SLA minimum in the tAUS presented in Fig. 2b for the period 2003-2022. The time series of amplitude of the SLA minimum is normalized prior to calculating the regressions (see methods). Regressions are calculated for time periods relative to the timing of the annual SLA minimum given in the titles (see methods). Significant correlation (95% confidence level) is marked by grey lines. The regression slopes in the maps correspond to a higher amplitude of the SLA minimum in the tAUS. The maps were created using the python packages matplotlib (version 3.8.4)[2] and basemap (version 1.4.1, https://matplotlib.org/basemap/stable/).

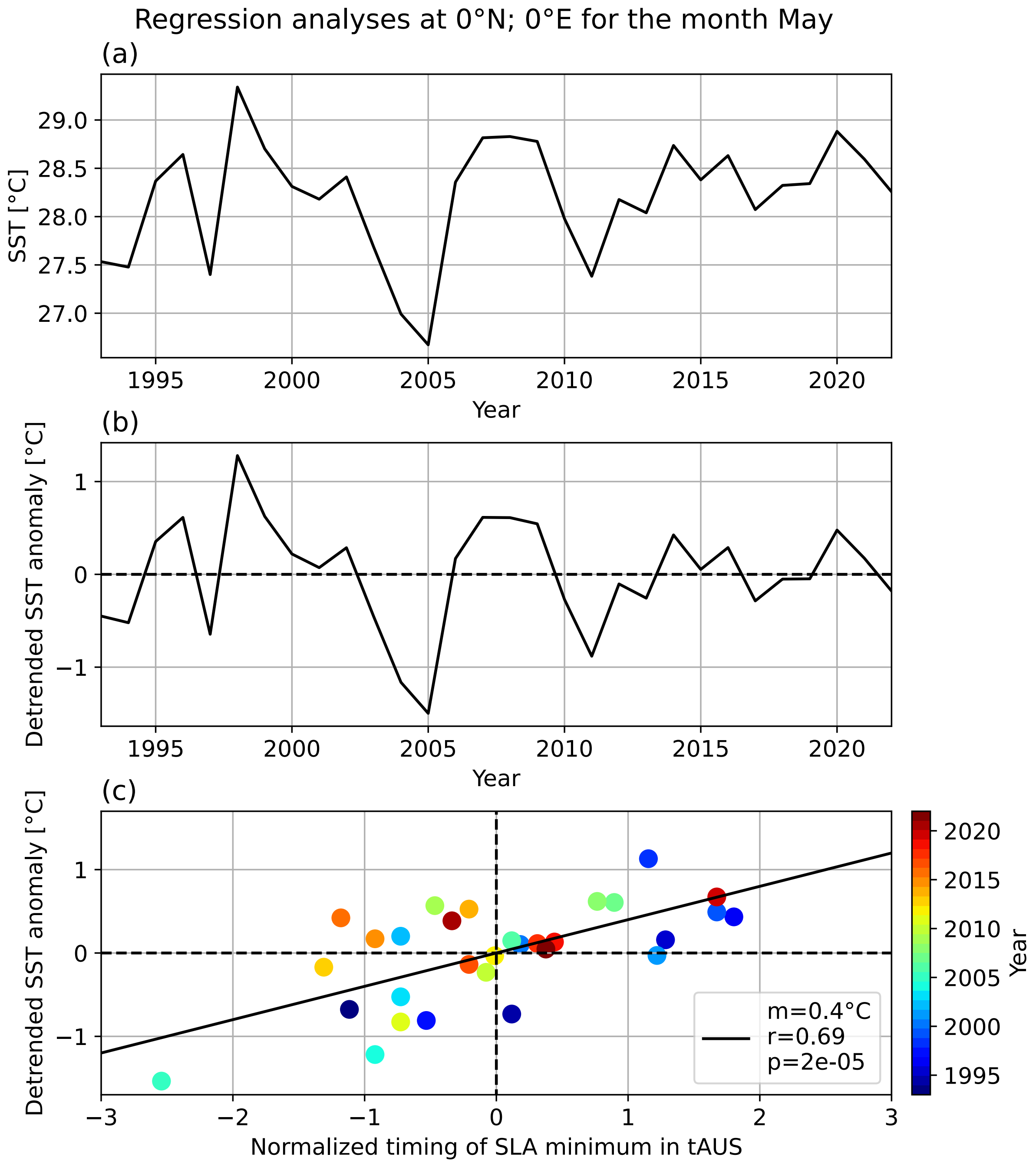

Figure S6: Regression analysis of SST at 0°N, 0°E in May onto the timing of the SLA minimum in the tAUS. **(a)** Time series of SST for May from 1993 to 2022. **(b)** Detrended and demeaned time series of SST as presented in (a). **(c)** Scatter plot of detrended and demeaned SST anomalies versus the normalized timing of the SLA minimum in the tAUS. Colors indicate the respective year of the data point. Linear regression line is shown in black. The slope of the regression, m, the Pearson correlation coefficient, r, and the p-value of the correlation, p, are given in the legend. Note that this example corresponds to the map shown in Fig. S1d.
